# Antioxidant Activities of Ethanolic Extracts Obtained from α-Pinene-Containing Plants and Their Use in Cosmetic Emulsions

**DOI:** 10.3390/antiox13070811

**Published:** 2024-07-04

**Authors:** Jadwiga Grzeszczak, Agnieszka Wróblewska, Adam Klimowicz, Sylwia Gajewska, Łukasz Kucharski, Zvi C. Koren, Katarzyna Janda-Milczarek

**Affiliations:** 1 Department of Catalytic and Sorbent Materials Engineering, Faculty of Chemical Technology and Engineering, West Pomeranian University of Technology in Szczecin, Piastów Ave. 42, 71-065 Szczecin, Poland; jadwiga.tolpa@zut.edu.pl (J.G.); gs54727@zut.edu.pl (S.G.); 2 Department of Cosmetic and Pharmaceutical Chemistry, Pomeranian Medical University in Szczecin, 72, Powstancow Wlkp St., 70111 Szczecin, Poland; lukasz.kucharski@pum.edu.pl; 3 Department of Chemical Engineering, Shenkar College of Engineering, Design and Art, 12 Anna Frank St., Ramat Gan 52526, Israel; zvi@shenkar.ac.il; 4 Department of Human Nutrition and Metabolomics, Pomeranian Medical University in Szczecin, 24 Broniewskiego St., 71-460 Szczecin, Poland; katarzyna.janda.milczarek@pum.edu.pl

**Keywords:** α-pinene, *Pinus sylvestris* L. cone, *Rosmarinus officinalis* leaves, *Salvia officinalis* L. leaves, antioxidant activity, DPPH, ABTS, emulsion

## Abstract

α-Pinene is the bicyclic, unsaturated terpene hydrocarbon present in many plants. Due to its beneficial chemical properties, this compound is of great interest and has found numerous applications as a raw material in many chemical industries as well as in medicine and cosmetics. The aim of this study was to evaluate the antioxidant activities of ethanolic extracts obtained from plants containing α-pinene and to test the properties of cosmetic emulsions prepared with these extracts. The raw plant materials consisted of fresh parts of *Pinus sylvestris* L., such as cones, needles, and branches, as well as dried unground and ground pinecones; dried and fresh *Rosmarinus officinalis* leaves; dried *Levisticum officinale* leaves; and dried *Salvia officinalis* L. leaves. The plant materials were individually extracted with 40% (*v*/*v*), 70% (*v*/*v*), and 96% (*v*/*v*) ethanol using ultrasound-assisted extraction (UAE) for 15, 30, or 60 min. This method is a green extraction technique, frequently applied to isolate active substances from plants. For the selected plant materials, Soxhlet extraction with 96% (*v*/*v*) ethanol was also performed. The qualitative and quantitative analyses of the components in the selected extracts were performed with gas chromatography coupled with mass spectrometry (GC-MS). The antioxidant activities of the extracts were evaluated with the DPPH and ABTS methods. The extracts of three plant materials with the highest antioxidant activities—dried *Rosmarinus officinalis* leaves, dried *Salvia officinalis* L. leaves, and dried and ground *Pinus sylvestris* L. cones—were selected to be incorporated in cosmetic emulsions containing glyceryl monostearate and Olivem 1000 as emulsifiers. The stabilities and antioxidant activities of the emulsions were evaluated. Moreover, the antimicrobial properties of the emulsions using microbiological tests were also determined. The findings suggest that the prepared emulsions are stable cosmetic products with a high antioxidant potential.

## 1. Introduction

α-Pinene is a bicyclic, unsaturated terpene hydrocarbon widely found in nature. Natural sources of α-pinene include coniferous trees, such as juniper [1], spruce [2], fir [3], and pine [4], and herbs and fruits. This monoterpene compound can be obtained from various parts of the pine tree, such as needles [5], branches [6], and cones [7], as well as from many herbs, such as sage [8], lovage [9], thyme [10], chamomile [11], and black pepper [12]. α-Pinene is the main component of rosemary oil with anticancer, anti-inflammatory, and antioxidant activity [13]. This monoterpene can be found in citrus fruits, such as tangerines [14], grapefruits [15], lemons [16], and oranges [17], as well as in other fruits such as watermelons [12] and walnuts [18]. This compound has a specific, fresh, pine-like scent [12,19]. Due to its valuable chemical properties, it is frequently used in the chemical and perfumery industries as well as in medicine [20].

α-Pinene exhibits a number of therapeutic properties. This compound has been shown to be active against Gram-positive and Gram-negative bacteria [21]. It was observed that it is effective in inhibiting ovarian and liver cancer [12]. Moreover, α-pinene has anti-inflammatory [22], antifungal [23], and antinociceptive properties [24] and exhibits anti-osteoarthritis, anti-ulcerogenic, and gastroprotective effects [5].

The antioxidant activities of ethanolic extracts or essential oils obtained from pinene-containing plants were described in several scientific reports. However, different methods of producing the extracts were used in these studies. Therefore, it is very difficult to compare the results obtained in these studies. An example of different processing methods is the research on rosemary. Christopolou et al. [25] studied the antioxidant activity of the essential oil obtained from fresh rosemary leaves. In that study, an ethanolic extract was prepared from 8-day old dried leaves, which were then subjected to maceration for 25 days in 40 mL of solvent containing 38% (*v*/*v*) ethanol at 20–25 °C. Both the essential oil and the extract exhibited good antioxidant activity, but rosemary essential oil was far more effective than the ethanolic extract [25]. A different processing method for the preparation of rosemary extracts is the work of Kamli et al. [26]. In these studies, the plant material was first dried in the shade at 30 ± 2 °C, then crushed in a blender, followed by filtering the powdered material using a sieve of about a 0.3 mm aperture size. This material was then individually extracted with each of these three organic solvents, ethyl acetate, ethanol, and water, for 48 h using a Soxhlet extractor. The extracted material was concentrated and was stored at −80 °C for future use. These studies showed that the antioxidant potential of the three extracts tested was in the following order: ethyl acetate > ethanol > aqueous [26].

The unique structure of α-pinene allows for this monoterpene compound to be used as a raw material in the synthesis of valuable organic compounds, which can be applied in the perfume, food, agricultural, and pharmaceutical industries. As an example, verbenone, one of the main products of α-pinene oxidation, is used as a cough suppressant and as an insect repellent. Camphor, derived indirectly from α-pinene, is applied as a flavoring, pharmaceutical, and cosmetic agent and as a plasticizer. Other α-pinene derivatives such as sabinene, thujone, thujene, and umbellulate are used as food flavorings, perfume ingredients, and antimicrobial agents [20].

An interest in emulsions and their applications has increased over the years, as they can be beneficial in many applications [27,28]. Emulsions contain aqueous and oil phases, and in order to obtain a stable emulsion, emulsifiers are added. Recently, substances of natural origin were used as emulsifiers. One such emulsifier is Olivem 1000, which consists of cetearyl olivate and sorbitan olivate [29], and emulsions containing natural active substances with this emulsifier were produced [28].

The use of ethanolic extracts obtained from α-pinene-containing plants to prepare emulsions using Olivem 1000 as the emulsifier has not been described in the scientific literature so far. Similarly, the antioxidant activities of emulsions obtained in this way was not tested. These are two directions of research that are new in our work compared to the current state of knowledge.

The purpose of the first stage of our research was to examine the possibility of using the ultrasound-assisted extraction (UAE) method to obtain ethanolic extracts from α-pinene-containing plants, such as *Pinus sylvestris* L., *Rosmarinus officinalis*, *Levisticum officinale*, and *Salvia officinalis*. These extracts were performed with 40% (*v*/*v*), 70% (*v*/*v*), and 96% (*v*/*v*) ethanol. The antioxidant activities of these extracts were evaluated with the DPPH and ABTS methods. To verify whether the selected plants contain α-pinene, the ethanol extracts were subjected to an analysis with the GC-MS method.

In the second stage, for selected plant materials, Soxhlet extraction with 96% (*v*/*v*) ethanol was also performed. Both ultrasound-assisted extraction and Soxhlet extraction are known as methods that lead to good recovery of the active compounds from the plant material. Additionally, UAE is a green technique with low solvent consumption, and the aim of our research was to assess the possibility of using this method compared to the more classic ones.

In the next phase of our study, emulsions containing ethanolic extracts with the highest antioxidant activities were prepared using glycerol monostearate and Olivem 1000 as emulsifiers, and their stabilities were evaluated. The aim of this investigation was to determine whether it would be possible to produce stable emulsions with Olivem 1000 as well as to compare the emulsifying abilities of Olivem 1000 and glycerol monostearate. The optimal amount of α-pinene extract that is to be added to obtain the stable emulsion was also evaluated. The antioxidant activities of the emulsions were studied, and microbiological tests were performed to determine their antimicrobial properties. The aim of this latter investigation was to investigate how ethanolic extracts containing α-pinene affect the presence of bacteria and fungi in these emulsions.

Our research is intended to constitute a scientific basis for emphasizing the importance of extracts containing α-pinene as ingredients with antioxidant properties and may constitute a starting point for the development of cosmetic preparations with high antioxidant activity.

## 2. Materials and Methods

### 2.1. Raw Materials

For the ultrasound-assisted extractions, the plant materials were individually extracted with 40% (*v*/*v*), 70% (*v*/*v*), and 96% (*v*/*v*) ethanol. The 40% (*v*/*v*) and 70% (*v*/*v*) ethanol solutions were prepared from 96% (*v*/*v*) ethanol (Stanlab, Lublin, Poland) via dilution with water. The following plant materials were used for the preparation of ethanolic extracts: fresh parts of *Pinus sylvestris* L., such as cones, needles, and branches, as well as dried unground and ground pinecones (Tanowo, Poland); dried and fresh *Rosmarinus officinalis* leaves (Rolpol Agra, Tarkowo Górne, Poland); dried *Levisticum officinale* leaves (Rolpol Agra, Tarkowo Górne, Poland); and dried *Salvia officinalis* L. leaves (Rolpol Agra, Tarkowo Górne, Poland).

**Figure 1:**
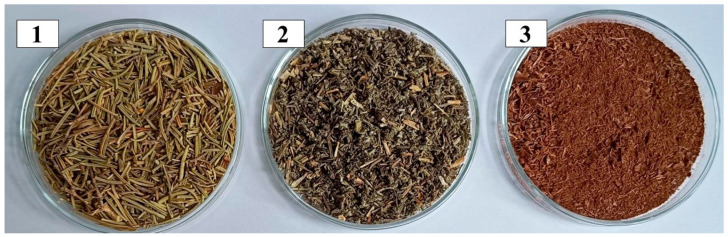
Raw plant materials selected for the studies: 1—dried *Rosmarinus officinalis* leaves, 2—dried *Salvia officinalis* L. leaves, 3—dried ground *Pinus sylvestris* L. cones.

Raw materials for the extraction of α-pinene using the Soxhlet apparatus consisted of the following (shown in Figure 1): dried *Rosmarinus officinalis* leaves (Rolpol Agra, Tarkowo Górne, Poland); dried *Salvia officinalis* L. leaves (Rolpol Agra, Tarkowo Górne, Poland); and dried and ground *Pinus sylvestris* L. cones (Tanowo, Poland). A concentration of 96% (*v*/*v*) ethanol (Stanlab, Lublin, Poland) was used to extract α-pinene from these raw materials.

The following components were used to prepare the emulsions: cetyl alcohol, lanolin, almond oil (all from Zrób sobie krem, Prochowice, Poland), and glycerin (Sigma Aldrich, Poznań, Poland). Glyceryl stearate (Zrób sobie krem, Prochowice, Poland) and Olivem 1000 (EcoSpa, Józefosław, Poland) were used as emulsifiers.

### 2.2. Ultrasound-Assisted Extraction of Plant Materials

The method of performing the ultrasonic-assisted extraction process was adopted based on the results of the research conducted and described in our previous works [30,31,32].

To prepare the extracts into glass tubes fitted with glass stoppers, 0.5 g of the appropriate plant material was placed, and then, 9.5 mL of solvent—40% (*v*/*v*), 70% (*v*/*v*), or 96% (*v*/*v*) ethanol—was added. Each set of 9 stoppered tubes containing one type of raw material and one of the solvents described above was subjected to ultrasound-assisted extraction (Polsonic, Poland working at a frequency of 40 kHz) for 15, 30, or 60 min, respectively. No heating was used during the extraction.

After the extraction, the contents of the tubes were filtered using filter strainers to separate the plant residues from the extract. The extracts were stored in tightly sealed glass vials in the dark at room temperature in the same solvent in which they were extracted until the antioxidant activity was determined.

Ultrasound-assisted extraction is a green extraction technique due to, among other factors, low solvent consumption, reduction of extraction time and energy consumption, safety, and environmental friendliness [33].

### 2.3. Extraction of Plant Materials Using Soxhlet Apparatus

A 100 mL Soxhlet apparatus, consisting of a 500 mL round-bottom flask, a reflux condenser, and a heating mantle, was used to prepare extracts from the following selected plant materials: dried *Rosmarinus officinalis* leaves, dried *Salvia officinalis* L. leaves, and dried and ground *Pinus sylvestris* L. cones. In this method, 10 g of the appropriate plant material was put into a thimble in the Soxhlet apparatus, then 200 mL of 96% (*v*/*v*) ethanol was added into the round-bottom flask, and the extraction was performed for 1 h. Extracts obtained using the Soxhlet method were subjected to further processing (i.e., filtration using filter papers) that was similar to those obtained using the ultrasound-assisted extraction method. We attempted to remove the solvent from the obtained extracts using an evaporator, but the yield of the process was very low; hence, we added dissolved extracts, not dry ones, to the emulsion.

### 2.4. Analysis of Ethanol Extracts by GC-MS

The ethanol extracts were subjected to qualitative and qualitative analysis with the gas chromatography coupled with mass spectrometry (GC-MS) method in order to identify the various components in the extracts [34].

The apparatus for this analysis consisted of a Trace 2000 gas chromatograph (ThermoQuest/CE Instruments, Milan, Italy) with a Voyager quadrupole mass spectrometer (ThermoQuest/Finnigan, MassLab Group, Manchester, UK) operated in electron impact ionization (EI+) mode. An Agilent J&W DB-5MS column with a length of 30 m, an inner diameter of 0.25 mm, and a layer thickness of 0.5 µm was used. Helium, grade 6 (Air Liquid, Szczecin, Poland), was used as the carrier gas at a constant flow rate of 1 mL/min. The temperature of the split/splitless injector was 200 °C. Sample volumes of 0.1 µL were injected manually into the injection port of the gas chromatograph by means of a 1-microliter syringe. The oven was maintained at 50 °C for 2.5 min, and then, the temperature was increased by 10 °C/min to 300 °C and maintained for 4 min. The temperature of the transfer line was 200 °C. The ionization occurred with a kinetic energy of the impacting electrons of 70 eV. Mass spectra were obtained in full-scan mode in the mass range *m*/*z* 35–500.

Identification of the individual compounds present in the extracts was performed by comparing their mass spectra with those of the standards from REPLIB. In all experiments, the spectra were examined manually in Qual Browser. Quantitative GC-MS analysis was performed using the surface normalization method. The relative content [%] of individual compounds in the extracts was determined using Xcalibur software version 1.2 as the percentage of the area of their peaks relative to the total peak area of all compounds in the chromatogram.

### 2.5. Antioxidant Characterizations of Ethanolic Plant Extracts via Spectrophotometric Methods

The antioxidant activities of ethanolic extracts obtained from plants containing α-pinene were evaluated with the spectrophotometric method based on DPPH and ABTS radical reduction, as previously described [35,36]. The absorbances at 517 nm (DPPH) and 734 nm (ABTS) wavelengths were measured using a Hitachi U-5100 Spectrophotometer. Activity was expressed as Trolox equivalent antioxidant capacity (TEAC) [mg Trolox/g].

The antioxidant activities of emulsions were evaluated via the spectrophotometric method based on DPPH radical reduction by measuring the absorbance at 517 nm. Activity was expressed as radical scavenging activity [%RSA] as follows:

$$\%RSA=\left(1-\frac{A_{t}}{A_{0}}\right)\times 100\%,$$

where *A_t_* is the absorbance of the tested sample and *A*_0_ is the absorbance of the control sample.

DPPH and ABTS calibration curves were prepared based on the relationship of %RSA versus Trolox concentration and the relationship of absorbance versus Trolox concentration. The Supplementary Equations (S1) and (S2) present the calibration curves.

#### Evaluation of the Antioxidant Activities of Ethanol Extracts Obtained from Plants Containing α-Pinene

- DPPH method

An aliquot of 2500 µL of working ethanolic DPPH radical solution (absorbance 1.00 ± 0.02 at 517 nm) was introduced into a spectrophotometric cuvette, and then, 132 µL of extract was added, and the contents were mixed. The incubation time was 10 min at room temperature, and after this time, absorbance measurements were taken at 517 nm. All the analyses were performed in 3 replicates, and the results were expressed as Trolox equivalent antioxidant capacity (TEAC) in mg/g [35,36].

- ABTS method

An aliquot of 2500 µL of 7 mM ABTS solution was introduced into a spectrophotometric cuvette, and then, 25 µL of extract was added. The contents of each cuvette were mixed by shaking and then incubated at room temperature for 6 min. After this time, the absorbance was measured at 734 nm. All the analyses were performed in 3 replicates, and the results were expressed as Trolox equivalent antioxidant capacity (TEAC) in mg/g [35,36].

### 2.6. Preparation of Emulsions with Ethanolic Plant Extracts

In the first step, a standard emulsion was prepared without extract. For this purpose, 6.00 g glyceryl stearate or 6.00 g Olivem 1000, 0.500 g cetyl alcohol, 1.00 g lanolin, and 2.00 g almond oil were introduced into the tube. The whole mixture was heated in a water bath at 80 °C until the ingredients dissolved. Next, 36.50 g of water and 1.50 g of glycerin were introduced into a 50 mL glass beaker, and here too, the whole mixture was heated in a water bath at 80 °C until the ingredients dissolved. Then, the oil phase was added to the water phase, and the mixture was mixed with a recipe mixer from Eprus at 1960 rpm for 5.45 min.

**Figure 2:**
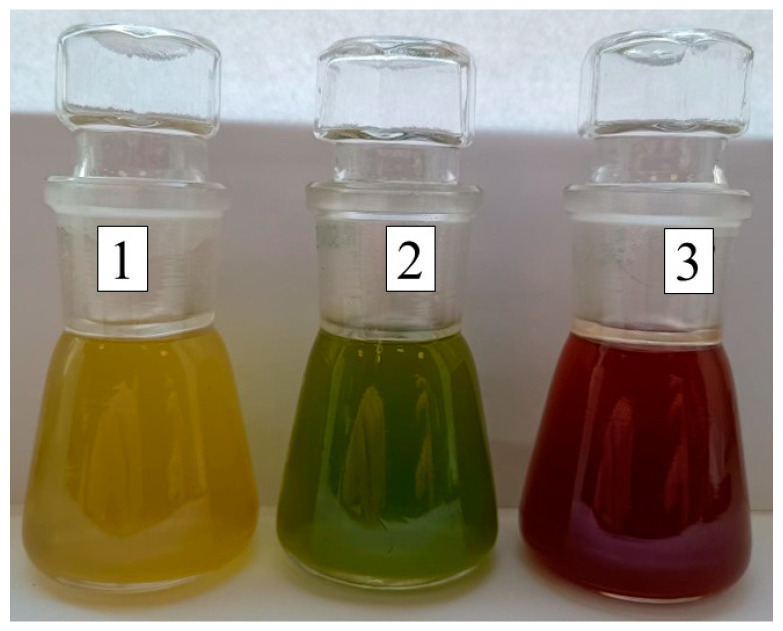
Ethanolic extracts: 1—dried *Rosmarinus officinalis* leaf extract, 2—dried *Salvia officinalis* L. leaf extract, 3—dried and ground *Pinus sylvestris* L. cone extract.

In the second stage, emulsions were prepared with the addition of extracts from three plant materials with the highest antioxidant activity, i.e., dried *Rosmarinus officinalis* leaf extract, dried *Salvia officinalis* L. leaf extract, and dried and ground *Pinus sylvestris* L. cone extract (Figure 2).

For this purpose, 6.00 g of glyceryl stearate emulsifier or 6.00 g of Olivem, 0.50 g of cetyl alcohol, 1.00 g of lanolin, and 2.00 g of almond oil were introduced into the tube. The whole mixture was heated in the water bath at 80 °C until the ingredients dissolved. Next, 12.75 g to 32.94 g water and 1.50 g glycerin were introduced into a 50 mL glass beaker. The beaker was heated in a water bath at 80 °C until the ingredients dissolved. The oil phase was added to the water phase, and the emulsion was cooled to 40 °C. Then the extracts were added in the following amounts: 7.5%; 10.0%; 12.5%; 15.0%; 17.5%; 20.0%; 22.5%; 25.0%; 27.5%; 30.0%; 40.0%; 50.0% to the total. The whole mixture was mixed using Eprus mixer at 1960 rpm for 5.45 min.

#### Evaluation of the Stabilities and pH of the Emulsions

To test the stabilities of the resulting emulsions, 5.0 g of emulsion was collected in the glass tube and centrifuged at a speed of 3500 rpm (1776× *g*).

The pH of each resulting emulsion was tested using a pH meter.

### 2.7. Testing the Antioxidant Activities of Emulsions

To test the antioxidant potential of the prepared emulsions, 1.0 g of emulsion was placed in the glass flask, then 9.0 g of acetone was added. The whole mixture was stirred at 500 rpm for 30 min. After this time, 2500 µL of working DPPH solution was added into the glass cuvette, then 132 µL of the mixture (emulsion or acetone) was added, and the content of each cuvette was mixed by shaking. Three samples were made for each mixture. The incubation time was 10 min at room temperature, and after this time, absorbance measurements were taken at 517 nm.

### 2.8. Testing the Antimicrobial Stabilities of Emulsions

The antimicrobial stabilities of selected emulsions with the highest antioxidant activities (the highest activity of the evaluated plant material) were determined using microbiological tests. These emulsions contained extracts of dried *Rosmarinus officinalis* leaves (amount of extract 22.5% and 25.0%), dried *Salvia officinalis* L. leaves (amount of extract 22.5%), dried ground *Pinus sylvestris* L. cones (amount of extract 22.5%), and the reference emulsion.

For microbiological tests, sterile plates with the medium applied on both sides were used. Two types of medium (*Mikrocount DUO*) were applied: the medium on the pink background was Rose Bengal Agar medium for yeast and mold determinations, and the yellow medium was *TTC* agar for incubation of bacteria.

In order to examine the antifouling stabilities of the selected emulsions, a very thin layer of emulsion was gently spread over the entire surface on both sides of the plate. The plate with the applied emulsion was then placed in a vial and the cap was tightly screwed on. The plates were then placed in an incubator heated to 30 °C for 4 days.

### 2.9. Statistical Analysis

Statistical analysis was performed using StatSoft Statistica 13.0 (STATISTICA 13.0; StatSoft Inc., Palo Alto, CA, USA) and Microsoft Excel 2021. Distributions of values for in-dividual parameters were analyzed using the Shapiro–Wilk test. Since the distribution of continuous variables differs from normal, the Kruskal–Wallis test was used to evaluate the differences between the studied parameters. Results were expressed as mean ± standard deviation (SD), and the statistical significance of the differences was determined based on median, upper quartile, and lower quartile. Spearman’s correlation test was used to determine the correlations between the parameters studied. Differences were considered significant at *p* ≤ 0.05.

## 3. Results and Discussion

### 3.1. Ultrasound-Assisted Extraction of Plant Materials

Ultrasound-assisted extraction was applied to obtain 81 ethanol extracts. The extracts varied in color depending on the plant material used, the concentration of the solvent, and the extraction time.

### 3.2. Extraction of Plant Materials Using Soxhlet Apparatus

As a result of extraction with a Soxhlet apparatus, as expected, three differently colored extracts were obtained depending on the plant material used.

### 3.3. Analysis of Ethanol Extracts by GC-MS

The presence of α-pinene in the ethanolic extracts was confirmed by the GC-MS analysis. In addition to α-pinene, compounds such as acetic acid, androstane-11, camphor, eucalyptol, verbenone, and others were determined in higher amounts. The detailed results of the GC-MS analyses are summarized in Supplementary Tables S1 and S2.

The main compound whose content in the extracts we were most interested in was α-pinene. The highest concentration of α-pinene in extracts obtained with ultrasound-assisted extraction was found for ethanolic extracts of dried *Rosmarinus officinalis* and *Levisticum officinale* leaves (14.5% and 9.6%, respectively). However, a comparison of extracts obtained with the two methods tested in this work (for dried *Rosmarinus officinalis* leaves, dried *Salvia officinalis* L. leaves, and dried and ground *Pinus sylvestris* L. cones) shows a significant reduction in the α-pinene content in extracts obtained in the Soxhlet apparatus. For the extract from dried *Rosmarinus officinalis* leaves, the α-pinene content decreased from 14.54% to 2.21% (almost a 7-fold reduction); for the extract from dried Salvia *officinalis* L. leaves, the content decreased from 4.83% to 2.85% (nearly a 2-fold reduction); and for the extract from dried and ground *Pinus sylvestris* L. cones, it decreased from 4.70 to 1.01% (almost a 5-fold reduction). The results presented in Supplementary Tables S1 and S2 indicate that the use of the Soxhlet extraction method was less effective. The reasons for this may be various: too short of an extraction time in the Soxhlet apparatus or a too-high temperature of the extraction medium in the Soxhlet apparatus, leading to the thermal decomposition of the extract components, mainly α-pinene (the boiling point of α-pinene is 155 °C [37]). Ultrasonic-assisted extraction was performed at an ambient temperature (approximately 25 °C, and no significant heating of the contents of the test tubes was observed during extraction). On the other hand, extraction in the Soxhlet apparatus was carried out at a temperature that was slightly lower than the boiling point of ethanol (the boiling point of ethanol is 78 °C [38]), as it was condensed in the cooler, which resulted in lowering its temperature.

To determine whether the extraction temperature in the Soxhlet apparatus may reduce the amount of α-pinene obtained (by causing the decomposition of this compound), we performed additional studies. In these studies, we extracted fresh pine needles in the Soxhlet apparatus using the two-stage extraction method. The α-pinene content in the extract obtained in this way was 20.39%. This method involved weighing about 10.0 g of plant material and placing it in the Soxhlet apparatus, and then, about 200 mL of 96% (*v*/*v*) ethanol was poured into the flask. After 6 h, the pine needles used in the extraction process were removed, another portion of fresh pine needles was weighed, and the extract obtained from the first stage of extraction served as the extraction agent in the second stage. In the second stage, extraction was also performed for 6 h. The results may therefore indicate that the extraction time using the Soxhlet apparatus in our comparative studies was most likely too short and that in future studies, this extraction time should be extended or even a multi-stage extraction should be used.

### 3.4. Evaluation of the Antioxidant Activities of Ethanolic Extracts Obtained from Plants Containing α-Pinene

#### 3.4.1. Extracts Prepared with Ultrasound-Assisted Extraction

- DPPH method

**Figure 3:**
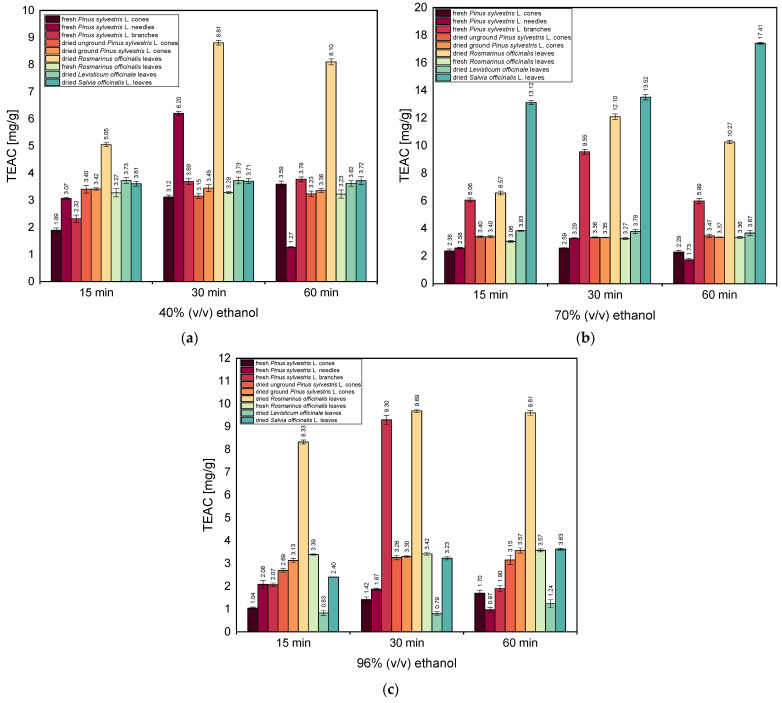
The antioxidant activities of ethanolic extracts in (**a**) 40% (*v*/*v*) ethanol, (**b**) 70% (*v*/*v*) ethanol, (**c**) 96% (*v*/*v*) ethanol, evaluated with the DPPH method. Vertical lines represent the standard deviation (SD).

Figure 3a–c show the antioxidant activities of ethanolic extracts in 40% (*v*/*v*), 70% (*v*/*v*), and 96% (*v*/*v*) ethanol, respectively, evaluated with the DPPH method.

Figure 3a shows that among the tested ethanolic extracts in 40% (*v*/*v*) ethanol, the highest antioxidant activities (5.05, 8.81, and 8.10 mg/g, for extraction times of 15 min, 30 min, and 60 min, respectively) were shown by the ethanolic extracts obtained from dried *Rosmarinus officinalis* leaves. Also noteworthy is the high TEAC value observed for fresh *Pinus sylvestris* L. needles—6.20 mg/g—after a 30 min extraction time. The TEAC values for extracts obtained from other raw plant materials were much lower and ranged from 1.89 to 3.78 mg/g.

Figure 3b shows that with 70% (*v*/*v*) ethanol, the highest TEAC values were observed for extracts obtained from dried *Salvia officinalis* L. leaves. For these extracts, the TEAC value increases with increasing extraction time from 13.12 mg/g (15 min) to 17.41 mg/g (60 min). It can also be seen from the figure that high values were obtained for extracts prepared with the dried *Rosmarinus officinalis* leaves; the TEAC value increased with increasing extraction time from 6.57 mg/g (15 min) to 12.10 mg/g (30 min), though there was a small decrease to 10.27 mg/g after 60 min, as was also the case with 40% (*v*/*v*) ethanolic extraction (Figure 3a). Thus, for this *Rosmarinus* dried plant material, a comparison of the antioxidant activity results obtained for extracts prepared with 40% (*v*/*v*) and 70% (*v*/*v*) ethanol shows that it is beneficial to perform the extraction for a maximum of 30 min. Additionally, it can be seen that for this plant material, increasing the concentration of ethanol used for extraction from 40% to 70% causes an increase of approximately 50% in the TEAC value for the extraction time of 30 min. Also noteworthy are the high TEAC values obtained for the extract from fresh *Pinus sylvestris* L. branches from 6.06 mg/g (15 min) to 9.55 mg/g (30 min).

Figure 3c shows that the extracts in 96% (*v*/*v*) ethanol obtained from dried *Rosmarinus officinalis* leaves have the highest antioxidant capacity, as was the case with the 40% ethanol extract. It can be observed that the TEAC value of the extract from this plant material remains at the level of 8.33–9.69 mg/g throughout the time range tested. For this plant material, using 96% ethanol yields nearly identical results for a 30 or 60 min extraction time. However, for 30 min extraction, this value is significantly lower than for extracts for this plant material obtained using 70% (*v*/*v*) ethanol. As in the case of extracts obtained from 70% (*v*/*v*) ethanol for 30 min extraction time, a high TEAC value (9.30 mg/g) was observed for the 96% ethanolic extract obtained from fresh *Pinus sylvestris* L. branches.

In summary, in almost all tests performed at this stage of our study, the highest TEAC values were generally found for extracts obtained from dried *Rosmarinus officinalis* leaves that were prepared for 30 min. Increasing the concentration of ethanol from 40% to 70%, used for the extraction of dried *Rosmarinus officinalis* leaves, increases the TEAC value to 12.10 mg/g for the extraction time of 30 min. The same ethanol concentration (70%) and the same extraction time (30 min) were also beneficial for the extraction of fresh *Pinus sylvestris* L. branches because the prolongation of the extraction time did not cause an increase in the TEAC values for this plant material. The use of ethanol at the concentration of 70% (*v*/*v*) was also beneficial to obtain the high values of TEAC for extracts from dried *Salvia officinalis* L. leaves. These were the highest TEAC values, 13.12 mg/g (15 min) to 17.41 mg/g (60 min), among those obtained for all extracts tested. For ethanol with other concentrations, such high TEAC values for this plant raw material were not observed.

- ABTS method

**Figure 4:**
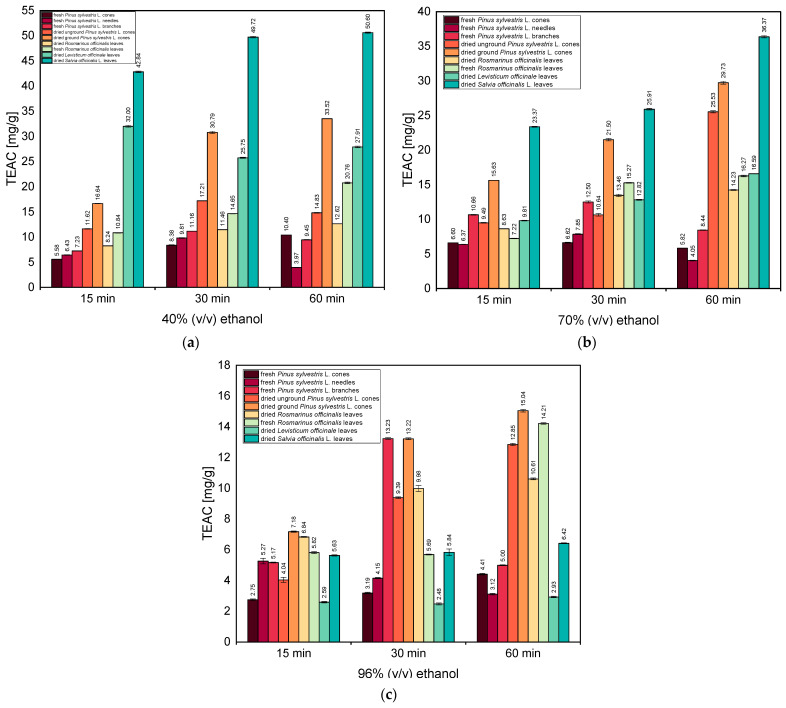
The antioxidant activities of ethanolic extracts in (**a**) 40% (*v*/*v*) ethanol, (**b**) 70% (*v*/*v*) ethanol, and (**c**) 96% (*v*/*v*) ethanol, evaluated with the ABTS method. Vertical lines represent the standard deviation (SD).

Figure 4a–c show the antioxidant activity of extracts in 40% (*v*/*v*), 70% (*v*/*v*), and 96% (*v*/*v*) ethanol, respectively, evaluated with the ABTS method.

From Figure 4a, it can be seen that among the ethanolic extracts tested, the one obtained from dried *Salvia officinalis* L. leaves after the extraction time of 60 min shows the highest antioxidant activity (50.60 mg/g). High values of TEAC were also observed for shorter extraction times for this plant material (42.84 mg/g for 15 min and 49.72 mg/g for 30 min). Additionally, high TEAC values were obtained for extracts from dried *Levisticum officinale* leaves (for 15 min—32.00 mg/g, for 30 min—25.75 mg/g, and for 60 min—27.91 mg/g). Similar TEAC values (excluding the extraction time of 15 min) were also obtained for dried ground *Pinus sylvestris* L. cones, 16.64 mg/g (15 min), 30.79 mg/g (30 min), and 33.52 mg/g (60 min).

Figure 4b shows that for extracts obtained with 70% (*v*/*v*) ethanol, the highest TEAC value (36.37 mg/g) was also obtained for dried *Salvia officinalis* L. leaves after the extraction time of 60 min. However, TEAC values obtained for this plant material were slightly lower than for studies with 40% (*v*/*v*) ethanol. The observed TEAC values for dried ground *Pinus sylvestris* L. cones were also very similar to values obtained for extraction with 40% (*v*/*v*) ethanol, 15.63 mg/g (15 min), 21.50 mg/g (30 min), and 29.73 mg/g (60 min). For the extraction time of 60 min, a high TEAC value was observed for another plant material—dried unground *Pinus sylvestris* L. cones (25.53 mg/g).

Figure 4c shows that the highest TEAC values were obtained for dried ground *Pinus sylvestris* L. cones. The TEAC values for this plant material increased from 7.18 mg/g (15 min) to 15.04 mg/g (60 min). Additionally, high values of TEAC were obtained for fresh *Rosmarinus officinalis* leaves and dried unground *Pinus sylvestris* L. cones (14.21 mg/g and 12.85 mg/g, respectively). Also noteworthy is the high TEAC value for fresh *Pinus sylvestris* L. branches obtained after the 30 min extraction (13.22 mg/g). In general, however, the TEAC values obtained during extraction with 96% (*v*/*v*) ethanol are lower than those obtained using 40% (*v*/*v*) or 70% (*v*/*v*) ethanol.

In summary, in almost all tests, the highest values of TEAC were observed for dried *Salvia officinalis* L. leaves and dried ground *Pinus sylvestris* L. cones. It was also observed that extraction with ethanol with the highest concentration caused the decrease in the values of TEAC for all studied plant materials.

#### 3.4.2. Soxhlet Extraction

During the studies on the antioxidant activities of the extracts obtained using the ultrasound-assisted extraction assessed with both the DPPH and ABTS methods, the ethanolic extracts obtained from dried *Rosmarinus officinalis* leaves showed the highest antioxidant activity. Moreover, it was observed that the antioxidant activities of the obtained ethanolic extracts increased in the following order: dried ground *Pinus sylvestris* L. cones < dried *Salvia officinalis* L. leaves < dried *Rosmarinus officinalis* leaves.

Since Soxhlet extraction is a standard technique used to obtain extracts, it was decided to compare the antioxidant activities of extracts obtained with this method with the extracts obtained using ultrasound-assisted extraction. Studies with a Soxhlet apparatus were performed for the three selected plant materials that were mentioned above. In the Soxhlet extraction, 96% (*v*/*v*) ethanol was applied.

**Figure 5:**
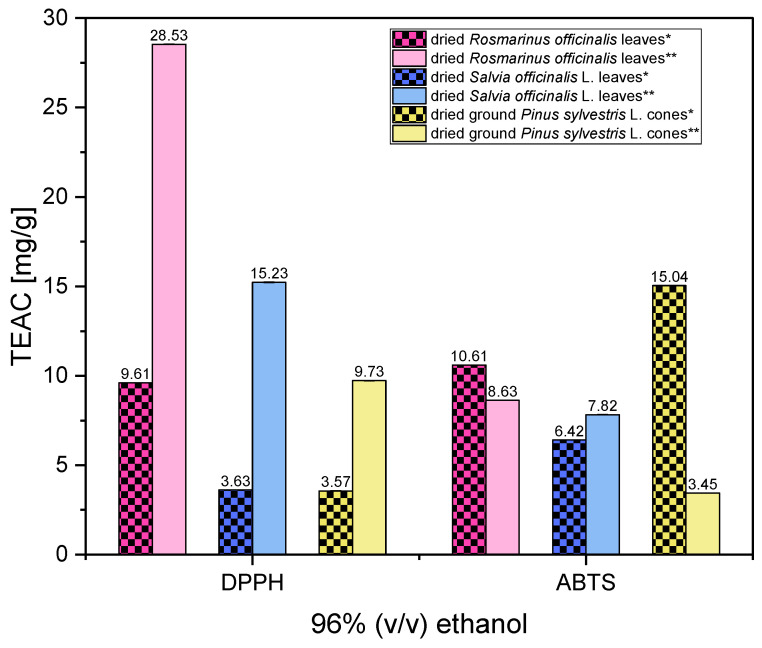
The antioxidant activities of ethanolic extracts in 96% (*v*/*v*) ethanol (60 min extraction time) obtained with the ultrasound-assisted extraction and the Soxhlet apparatus and evaluated with the DPPH and ABTS methods. Vertical lines represent the standard deviation (SD). * ultrasound-assisted extraction; ** the Soxhlet apparatus.

Figure 5 shows the antioxidant activities of extracts in 96% (*v*/*v*) ethanol obtained with the Soxhlet apparatus and evaluated with the DPPH and ABTS methods.

Figure 5 shows that for extracts obtained in the Soxhlet apparatus, higher TEAC values were obtained for the DPPH method than for the ABTS method. This is a different result from that obtained using ultrasound-assisted extraction. For example, in the case of dried *Rosmarinus officinalis* leaves, these values for the DPPH method are more than three times higher. At the same time, it can be observed that the highest TEAC value was obtained for extracts obtained for dried *Rosmarinus officinalis* leaves, and this conclusion is similar to that from studies performed with ultrasound-assisted extraction, described earlier. A comparison of the results obtained at this stage of the studies with the results shown in Tables S1 and S2 shows that in the case of dried *Rosmarinus officinalis* leaves, there was a significant reduction in the α-pinene content from 14.5% (ultrasound-assisted extraction) to 2.21% (extraction in the Soxhlet apparatus); however, the extract still showed very good antioxidant properties—significantly higher in the DPPH method than the extract obtained using ultrasonic-assisted extraction. This means that other compounds present in this extract also influence its antioxidant effect. These compounds can be camphor and eucalyptol, whose antioxidant effects were also described in the scientific literature [39,40]. A comparison of the content of these compounds in extracts from dried *Rosmarinus officinalis* leaves shows that the concentration of these two compounds also decreased significantly in the extract obtained using the Soxhlet apparatus. However, new compounds, such as verbenol, were also determined in this extract. The antioxidant properties of verbenol have been described in the scientific literature [41]. However, determining which components of the extract have such a strong antioxidant effect requires further research.

In summary, it can be concluded that ethanolic extracts obtained from plants containing α-pinene show different antioxidant activities, which depend mainly on the type of plant material, solvent concentration (ethanol), extraction time, method of extraction and method applied to evaluate the antioxidant activity of the extract (a summary table is given as Supplementary Table S3). Taking into account the above-mentioned parameters, the extract of dried *Salvia officinalis* L. leaves obtained with 40% (*v*/*v*) ethanol for 60 min using ultrasound-assisted extraction and evaluated with the ABTS method shows the highest antioxidant activity among the tested extracts. The next important conclusion that can be drawn from the research presented above is that ultrasound-assisted extraction as the method of obtaining extracts with high antioxidant capacity is a very effective method. It allows for obtaining such extracts in a short time and at a relatively low temperature, which allows for the reduction of the costs associated with, for example, conducting the continuous extraction process using the Soxhlet method at the boiling point of the extraction medium. In our future research, it would be necessary to determine which of the compounds present in the extracts, apart from α-pinene, can significantly influence the antioxidant activity of the extracts. Compounds such as camphor, eucalyptol, and verbenol should be taken into account.

### 3.5. Comparison with Other Studies

Several studies were performed to evaluate the antioxidant activities of the extracts obtained from plants containing α-pinene. However, the methods of obtaining extracts and establishing the antioxidant activity presented in these studies were different from the methods applied in our study. Therefore, it is very difficult to compare our results with those previously described in the scientific literature. Below, we present the most important literature reports regarding the determination of the antioxidant activities of extracts obtained from plant materials containing α-pinene and the description of the methods applied to obtain extracts.

In the work of Christopoulou et al. [25], the ethanolic extract from rosemary was obtained from leaves dried for 8 days, which then were subjected to maceration for 25 days in 40 mL of solvent containing 38% (*v*/*v*) ethanol at 20–25 °C. In comparison with the ultrasound-assisted extraction method used in our study, this method required a very long extraction time, as much as 25 days. On the other hand, the preparation of our extracts took a relatively short time, maximum 60 min. The antioxidant activities of the ethanolic rosemary extracts were tested with DPPH, ABTS, and FRAP assays. Using the first method, the free radical scavenging activity, expressed in mg BHT/mL, was in the range of 1.04 ± 0.06 mg/mL. The ferric-reducing power evaluated with the FRAP method expressed in mg FeSO_4_/mL was 0.52 ± 0.05 mg/mL. The antioxidant activity determined with the ABTS method was in the range of 0.25 ± 0.02 mg Trolox/mL [25].

Kamli et al. [26] also studied the antioxidant activities of rosemary extracts, which they prepared as described in the Introduction above. The powdered plant material was extracted with three organic solvents, ethyl acetate, ethanol, and water, for 48 h using a Soxhlet extractor. The extracted material was concentrated and was stored at −80 °C for future use. The method of obtaining extracts used in their work is therefore very time-consuming, and such long extraction times at elevated temperatures may lead to changes in the structure of biologically active compounds as a result of thermal decomposition, isomerization, oxidation, or oligomerization. The antioxidant potential of the three extracts tested was found to be in the order of ethyl acetate > ethanol > aqueous, with IC50 values (the required concentration of extract sample for scavenging of 50% DPPH free radicals) of 272 µg/mL, 387 µg/mL, and 534 µg/mL, respectively. Moreover, the total phenolic content of various rosemary extracts was estimated with the Folin–Ciocalteu method and was represented as gallic acid equivalents (GAE/g extract). A significant amount of the phenolic content of 804 GAE /g extracts was found in ethyl acetate extract, followed by ethanolic (473 GAE/g) and aqueous (273 GAE/g).

Saini et al. studied the antioxidant activity of the ethanolic extract of rosemary leaves with the DPPH and ABTS methods. The higher antioxidant activity of 70 ± 4.67 μg/mL was obtained when the second method was used. In contrast, the antioxidant activity evaluated with the DPPH method was in the range of 40.76 ± 2.81 μg/mL. In their work, the extract for antioxidant tests was prepared in the following way: rosemary leaves were dried at 50 °C for 12 h followed by grinding and sieving. Powdered leaves were extracted with 70% ethanol for 24 h at 40 °C. The extract was collected and concentrated under reduced pressure, and the semisolid mass was dried overnight at 50 °C to obtain the dried extract. The extracts were reconstituted with the same solvent as used for the extraction to obtain 5% solutions and stored at 4 °C [42]. The method of preparing the extract for antioxidant tests presented in their work is very complicated and time-consuming compared with ultrasound-assisted extraction. Their extraction process lasted 24 h, followed by drying the extract and then diluting it for antioxidant tests.

Mokhtari et al. evaluated the antioxidant potential of the methanolic sage extract with the DPPH and FRAP methods. The antioxidant activity of this extract measured with the DPPH method (EC50—extract concentration reducing the absorbance of DPPH by 50%) was 77.21 ± 2.61 μg-mL^−1^, whereas the ferric reduction potential measured with FRAP was 69.21 ± 2.61 mM Fe(II) mg^−1^ extract. In this work, the air-dried sage was ground, and next, its extract was obtained using the ultrasound-assisted extraction procedure [19], where 100 g of each ground sample was mixed with 500 mL of methanol (70%) and the mixture was sonicated in the ultrasonic bath. The solvent excess was evaporated, and the concentrated extracts were stored and freeze-dried at −18 °C [43]. In their research, the authors used ultrasound-assisted extraction, as was performed in our study, but they used methanol, which is an unfit extractant for subsequent cosmetic applications of extracts. Additionally, a significantly larger amount of plant material and solvent were used: 200 times more plant material and about 50 times more solvent. For this reason, this method of obtaining extracts seems to be less beneficial compared with our technique.

Abdelkader et al. used the DPPH method to evaluate the antioxidant activity of the methanolic extract from *Salvia officinalis* leaves. The antioxidant activity of the extract was 130.56 ± 0.87 µg/mL [44]. Their method for obtaining extracts for antioxidant studies was complicated. The leaves of *Salvia officinalis* L. were dried immediately after harvesting in a shady place over a two-week period. Next, they were packed in paper bags and kept in the dark, and after that, the leaves were crushed using the house blender. The fresh aerial part of *Salvia officinalis* L. material was subjected to hydrodistillation for approximately 4 h in a Clevenger-type apparatus. The distilled essential oils were dried over anhydrous sodium sulfate and stored in tightly closed dark vials at 4–6 °C before analysis. The diluted working solutions of the test extracts were prepared in methanol.

In the work of Farhat et al. [37], the antioxidant activities of methanolic extracts of *Salvia officinalis* were evaluated with the DPPH, ABTS, and FRAP methods. The following antioxidant activity results were obtained: 16.91 ± 0.44 μg/mL (DPPH method), 318.62 ± 14.40 μM TE/mg (ABTS method), and 180.56 ± 19.30 mM Fe(II)/mg (FRAP method) [45]. Also in this case, the method used for obtaining extracts for antioxidant studies was complicated. In their work, the plant material was dried at room temperature until it reached a constant weight. Aerial parts of each sample were subjected to hydrodistillation for 3 h using the Clevenger-type apparatus. The oil obtained was separated from water, dried over anhydrous sodium sulfate, and kept in amber vials at 4 °C. The distilled plant material was dried at 35 °C until it reached a constant weight and then finely ground to pass a 2 mm sieve. For the extraction, dried samples of 0.5 g were first homogenized with 30 mL of petroleum ether under magnetic stirring for 5 min and dried at room temperature. Second, the samples were extracted using 150 mL of methanol in a Soxhlet extractor for 2 h under a nitrogen atmosphere.

Venkatesan et al. [38] studied the antioxidant activities of ethanol extracts obtained from pine needles. The dried needles were ground in the laboratory blender into fine powder. About 10 g of needle powder was extracted with different ratios of ethanol to distilled water at 60 °C for 15 h. The ethanolic extract obtained from *Pinus densiflora* needles with 20% (*v*/*v*) ethanol showed the antioxidant activity measured with the ABTS method of 9.02 ± 0.55 μg/mL and with the DPPH method of 83.70 ± 6.22 μg/mL. These studies showed that the concentration of ethanol used for the extraction greatly affects the extract’s antioxidant activity [46]. The conclusion presented in the above work agrees with that of our research reported in this article. However, in our studies, the most favorable ethanol concentration was 40% (*v*/*v*) or 70% (*v*/*v*), depending on the method used to measure antioxidant capacity.

Four methods, namely ABTS, DPPH, FRAP, and CUPRAC (CUPric Reducing Antioxidant Capacity), were used to test the antioxidant activity of the ethanolic extract obtained from *Scots pine*. The extract was obtained with a very time-consuming method in which the fresh green cones were cut into small pieces and the plant material was extracted using 5 times the volume of 90% ethanol under continuous mixing (250 rpm, 25 °C). Extraction was performed at 25 °C in the dark for 10 days. The following antioxidant activity results were obtained: 8.56 ± 0.43 mg/mL (ABTS method), 13.82 ± 0.69 mg/mL (DPPH method), 11.27 ± 0.56 mg/mL (FRAP method), and 10.90 ± 0.55 mg/mL (CUPRAC method) [47].

Šarac et al. examined the antioxidant activity of the methanolic extract of essential oil obtained from *P. nigra* ssp. *nigra* using the DPPH and ABTS methods. Using the first method, the antioxidant activity was 25.60 mg/mL, and for ABTS, the activity was 0.58 ± 0.005 mg/mL [48]. The essential oil was obtained with the hydrodistillation method. As in the case of the works cited above, this method is much more complicated compared with the ultrasound-assisted extraction method that we propose in this work in order to obtain extracts with high antioxidant capacity.

### 3.6. Statistical Analysis

Our intention was to demonstrate that in this type of work, tests indicating the significance of differences between data are commonly used for statistical inference. First, it is necessary to check whether the results obtained during the study have a distribution that follows a normal distribution. For this purpose, a test such as the Shapiro–Wilk test is used. The null hypothesis for this test assumes that our research sample comes from a population with a normal distribution. If the Shapiro–Wilk test reaches statistical significance (*p* < 0.05), this indicates a distribution that deviates from the Gaussian curve.

Depending on the Shapiro–Wilk test-checked distribution of the data, parametric tests, e.g., the Student’s *t*-test; one-way analysis of variance (ANOVA) (when the results obtained do not deviate from a normal distribution); or non-parametric tests, e.g., Wilcoxon test, Mann–Whitney test (when the results obtained deviate from a normal distribution), are used.

In our study, the distribution of results deviated from the normal distribution (*p* < 0.05), so non-parametric tests were used. The Kruskal–Wallis test [41,42], which is the equivalent of the ANOVA test in parametric testing, was used to assess the statistical significance of differences between data.

Descriptive statistics of antioxidant potentials measured with the DPPH and the ABTS methods are presented in the Supplementary Materials as Supplementary Tables S4 and S5, respectively.

Table 1 shows statistically significant differences between the antioxidant potentials evaluated with the DPPH method of the extracts shown in Figure 3, Figure 4 and Figure 5.

**Figure 6:**
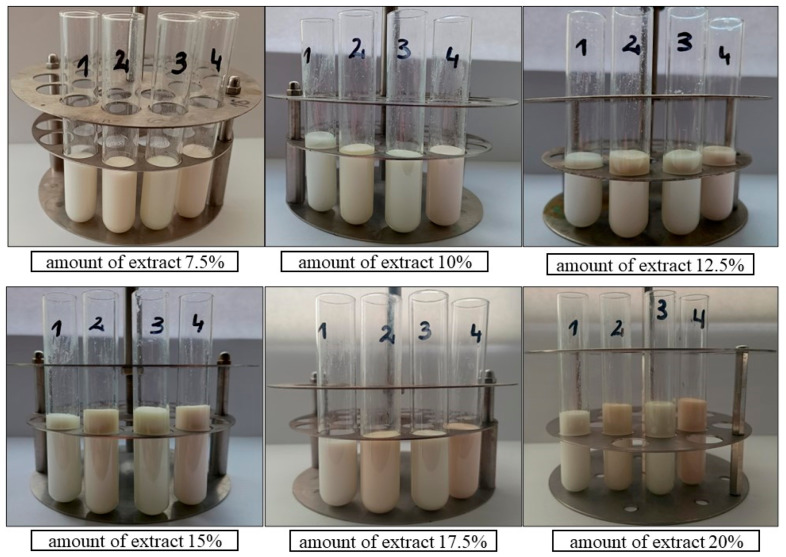
Emulsions containing Olivem 1000 as emulsifier: 1—base emulsion (without extract). With 7.5%, 10%, 12.5%, 15%, 17.5%, and 20% ethanolic extracts of the following plant materials: 2—dried *Rosmarinus officinalis* leaves, 3—dried *Salvia officinalis* L. leaves, 4—dried ground *Pinus sylvestris* L. cones.

**Figure 7:**
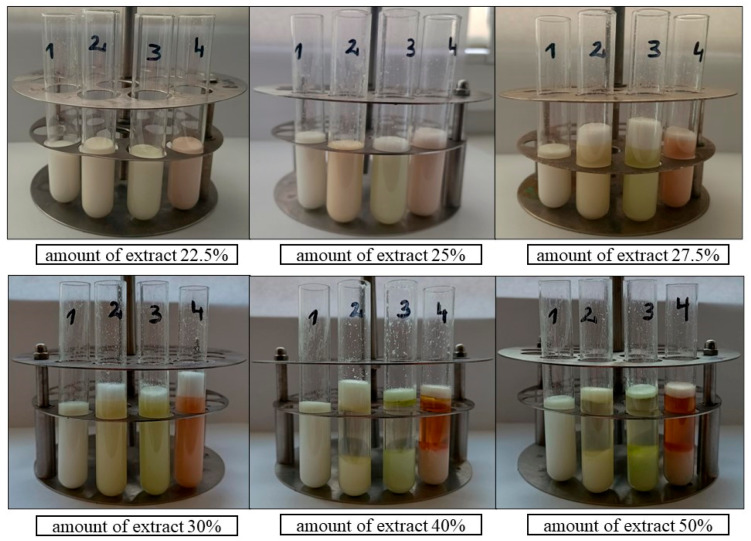
Emulsions containing Olivem 1000 as emulsifier: 1—base emulsion (without extract). With 22.5%, 25%, 27.5%, 30%, 40%, and 50% ethanolic extracts of the following plant materials: 2—dried *Rosmarinus officinalis* leaves, 3—dried *Salvia officinalis* L. leaves, 4—dried ground *Pinus sylvestris* L. cones.

**Figure 8:**
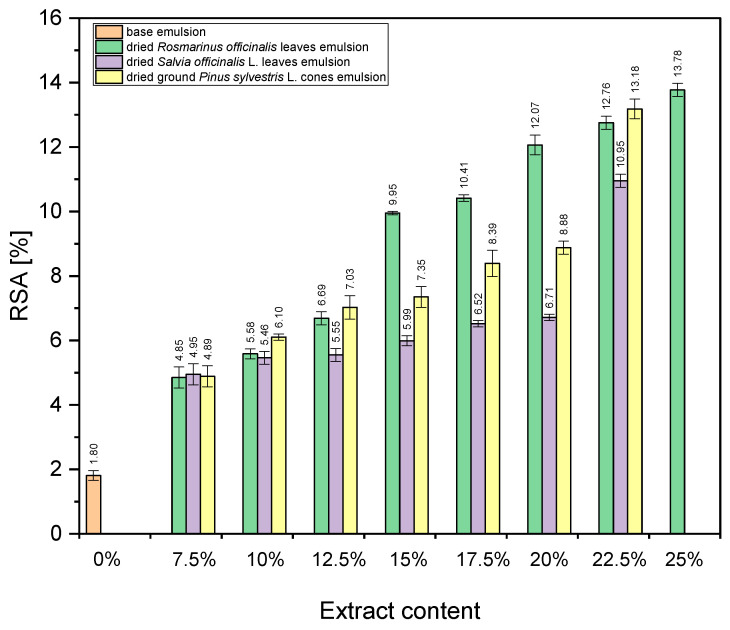
The antioxidant activities of the emulsions evaluated with the DPPH method and expressed as radical scavenging activity (%RSA). Vertical lines represent the standard deviation (SD).

Table 2 shows statistically significant differences between the antioxidant potential values of the extracts shown in Figure 6, Figure 7 and Figure 8.

The statistical analysis showed positive correlations between the antioxidant potentials measured with the DPPH and ABTS methods. In addition, the higher the ethanol concentration, the stronger the correlation was found (for 40% r = 0.53, for 70% r = 0.69, for 96% r = 0.87). There was also a statistically significant, strong, positive correlation (r = 0.880) between the concentration of the extract in the emulsion and the emulsion’s pH value. This means that as the concentration of the plant extract in the emulsion increased, its pH value increased.

### 3.7. Preparation of Emulsions with Ethanolic Extracts from Plants Containing α-Pinene

For the preparation of emulsion extracts, the plant materials with the highest antioxidant activity were selected for further study. The following materials were included: dried *Rosmarinus officinalis* leaves, dried *Salvia officinalis* L. leaves, and dried and ground *Pinus sylvestris* L. cones.

#### The Stability and pH of the Emulsions

Emulsions containing glycerol monostearate as an emulsifier were unstable. Supplementary Figure S2 shows these emulsions.

Emulsions containing Olivem 1000 as an emulsifier were prepared without an extract and with the addition of the following varying amounts of ethanolic extracts: 7.5%; 10%; 12.5%; 15%; 17.5%; 20%; and 22.5%. All these emulsions, without and with the extracts, were stable (Figure 6 and Figure 7).

Figure 8 shows that the emulsion with the addition of dried *Rosmarinus officinalis* leaf extract in the amount of 25% was stable, while emulsions with the addition of the other two extracts in the same amount (25%) were unstable. Emulsions with the amount of extract above 25%, i.e., 27.5%, 30%, 40%, and 50%, were unstable regardless of the raw material used.

Supplementary Figure S1 shows in detail emulsions containing Olivem 1000 as the emulsifier and different amounts of extracts prepared for our studies.

Supplementary Table S6 shows the pH values of the prepared emulsions. The results presented in this table show that the pH values of the emulsions were between 4.63 and 5.51. It can be observed that as the amount of added extract increases, the pH of the emulsions also increases (a strong correlation was found: r = 0.880).

### 3.8. Testing the Antioxidant Activities of Emulsions

Figure 8 shows the antioxidant activities of the obtained emulsions.

It can be observed from Figure 8 that increasing the amount of the added extract of dried *Rosmarinus officinalis* leaves increases the RSA of the emulsion from 4.85% (amount of extract 7%) to 13.78% (amount of extract 25%). With the increase in the amount of added extract from dried *Salvia officinalis* L. leaves, the RSA of the emulsion increases from 4.95% (amount of extract 7%) to 10.95% (amount of extract 22.5%). Similarly, increasing the amount of added extract from dried ground *Pinus sylvestris* L. cones increases the RSA of the emulsion from 4.89% (7% extract amount) to 13.18% (22.5% extract amount). The RSA of the base emulsion (without the addition of the extract) is 1.8%. Among the emulsions tested, the one with the addition of dried *Rosmarinus officinalis* leaf extract at 25% has the highest antioxidant activity (13.78%), while the base emulsion without the addition of an extract has the lowest antioxidant activity (1.80%).

In the case of concentrations of 7.5% and 10%, there were no statistically significant differences in the antioxidant potentials of the emulsions from different plant materials that were used to obtain the extract. In other cases (concentrations from 12.5% to 22.5%), it was found that there were statistically significant differences between the lowest and highest values of antioxidant potential (*p* ≤ 0.05). These results indicate that extracts from dried *Rosmarinus* leaves and dried unground *Pinus* cones at these concentrations were equally valuable.

### 3.9. Antimicrobial Stabilities of the Emulsions

**Figure 9:**
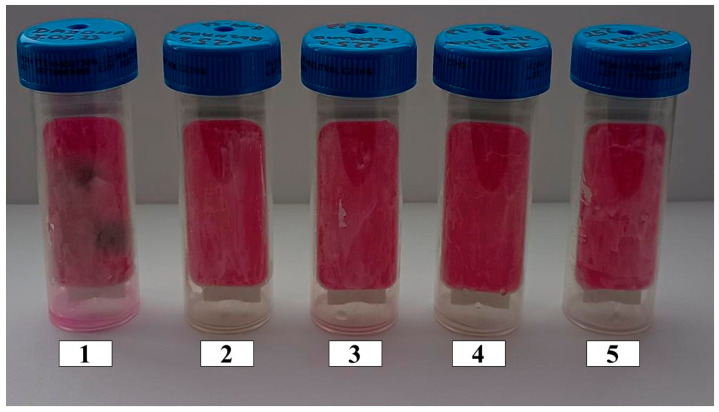
Microbiological plates with a medium for the determination of yeast and mold coated with emulsions: 1—base emulsion (without extract). Emulsions with a 22.5% ethanolic extract of the following plant materials: 2—dried *Rosmarinus officinalis* leaves, 3—dried *Salvia officinalis* leaves, 4—dried and ground *Pinus sylvestris* L. cones. 5—Emulsion with a 25% ethanolic extract of dried *Rosmarinus officinalis* leaves.

**Figure 10:**
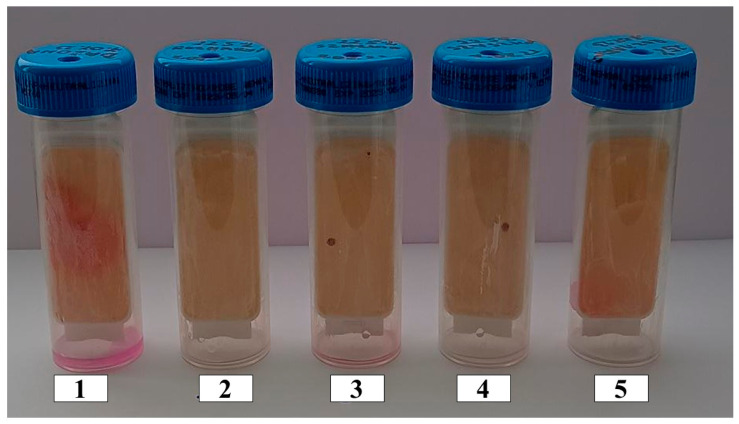
Microbiological plates with a substrate for the determination of bacteria coated with emulsions: 1—base emulsion (without extract). Emulsions with a 22.5% ethanolic extract of the following plant materials: 2—dried *Rosmarinus officinalis* leaves, 3—dried *Salvia officinalis* leaves, 4—dried and ground *Pinus sylvestris* L. cones. 5—Emulsion with a 25% ethanolic extract of dried *Rosmarinus officinalis* leaves.

The antimicrobial stabilities of the selected emulsions with the highest antioxidant activity of a given plant material were determined with microbiological tests. These emulsions consisted of extracts of dried *Rosmarinus officinalis* leaves (amount of extract 22.5% and 25%), dried *Salvia officinalis* L. leaves (amount of extract 22.5%), dried ground *Pinus sylvestris* L. cones (amount of extract 22.5%), and the base emulsion. The results of the microbiological tests are shown in Figure 9 and Figure 10.

Fungi appeared only on the plate coated with the base emulsion without the addition of the extract. No yeast or mold was observed on the other plates, which were coated with emulsions with added extracts.

A few bacteria, in the form of a pinkish-red colony, appeared on the plate coated with the emulsion with the 22.5% extract from dried *Salvia officinalis* leaves and on the plate coated with the emulsion with the 22.5% extract from dried and ground *Pinus sylvestris* L. cones. The amount of bacteria observed on the plates is less than 1.000 C.F.U/mL (1.000 bacteria per milliliter) and is acceptable for the emulsions obtained. The part of the plate coated with the base emulsion has a uniform red color and shows numerous pink-red colonies forming a cluster of bacteria.

Based on the microbiological tests, it can be concluded that all emulsions with the addition of ethanolic extracts obtained from plants containing α-pinene show excellent antimicrobial stability.

## 4. Conclusions

Our research conducted with plant extracts containing α-pinene can be the scientific basis for future applications of these extracts in the cosmetics industry and in medicine. Our work fits perfectly into the new trends in modern cosmetic preparations related to the use of biologically active compounds, including preservatives and antioxidants, from natural sources instead of synthetic compounds. With the use of these bioactive components extracted from plant materials, modern cosmetic products meet such requirements in the use of renewable raw materials and the application of sustainable cosmetic-production technologies. We have shown that it is very important to select the appropriate method for the extraction of the biologically active substances from plant material as well as the selection of the appropriate plant source of these substances.

This study demonstrates that the ultrasound-assisted extraction method can be used as a tool to obtain extracts containing α-pinene with a high antioxidant activity. Furthermore, our studies on the preparation of emulsions with these extracts showed that the application of Olivem 1000 as the emulsifier made it possible to create stable cosmetic emulsions exhibiting high antioxidant activity. Microbiological tests of these emulsions yielded the acceptable limited amount of microorganisms in the resulting preparations and that these extracts can reduce the degree of microbiological contamination in cosmetic preparations. It may suggest the possibility of using extracts containing α-pinene as the potential ingredients of anti-aging cosmetics. However, refining the optimal composition of the cosmetic product requires further research.

## Figures and Tables

**Table 1:** Statistically significant differences between the antioxidant potentials of the extracts measured with the DPPH method (at *p* ≤ 0.05).

Ethanol Concentration [% (*v*/*v*)]	Extraction Time (min)		
	15	30	60
40	- dried *Rosmarinus leaves* vs. fresh *Pinus* cones	- dried *Rosmarinus leaves* - vs. fresh *Pinus* cones - dried *Rosmarinus leaves* - vs. dried unground *Pinus* cones	- dried *Rosmarinus* leaves vs. fresh *Pinus* needles
70	- dried *Salvia* leaves vs. fresh *Pinus* cones - dried *Salvia* leaves vs. fresh *Pinus* needles	- fresh *Pinus* cones - vs. dried *Rosmarinus leaves* - fresh *Pinus* cones vs. dried *Salvia* leaves	- dried *Rosmarinus leaves* vs. fresh *Pinus* needles - dried *Salvia* leaves vs. fresh *Pinus* cones - dried *Salvia* leaves vs. fresh *Pinus* needles
96	- dried *Rosmarinus leaves* vs. fresh *Pinus* cones - dried *Levisticum* leaves vs. dried and fresh *Rosmarinus leaves*	- dried *Levisticum* leaves vs. fresh *Pinus* branches - dried *Levisticum* leaves vs. dried *Rosmarinus leaves*	- dried *Rosmarinus leaves* vs. fresh *Pinus* needles - dried *Rosmarinus leaves* vs. dried *Levisticum* leaves

**Table 2:** Statistically significant differences between the antioxidant potential values of the extracts measured with the ABTS method (at *p* ≤ 0.05).

Ethanol Concentration [% (*v*/*v*)]	Extraction Time (min)		
	15	30	60
40	- dried *Salvia* leaves vs. fresh *Pinus* cones - dried *Salvia* leaves vs. fresh *Pinus* needles	- dried *Salvia* leaves vs. fresh *Pinus* cones - dried *Salvia* leaves vs. fresh *Pinus* needles - fresh *Pinus* cones. vs. dried unground *Pinus* cones	- dried *Salvia* leaves vs. fresh *Pinus* needles - dried unground *Pinus* cones vs. fresh *Pinus* needles
70	- dried *Salvia* leaves vs. fresh *Pinus sylvestris* cones - dried *Salvia* leaves vs. fresh *Pinus* needles	- dried unground *Pinus* cones vs fresh *Pinus* cones - fresh *Pinus* cones vs. dried *Salvia* leaves - dried *Salvia* leaves vs. fresh *Pinus* needle	- dried *Salvia* leaves vs. fresh *Pinus* cones - dried *Salvia* leaves vs. fresh *Pinus* needles
96	- dried *Levisticum* leaves vs. dried unground *Pinus* cones	- dried *Levisticum* leaves vs. fresh *Pinus* branches - dried *Levisticum* leaves vs. dried unground *Pinus* cones	- dried unground *Pinus* cones vs. fresh *Pinus* needle - dried unground *Pinus* cones vs. dried *Levisticum* leaves

## Data Availability

The data presented in this study are available on request from the corresponding authors.

## Supplementary Materials

The following supporting information can be downloaded at https://www.mdpi.com/article/10.3390/antiox13070811/s1, Table S1: GC-MS determinations of ethanolic extracts (with ultrasound-assisted extraction); Table S2: GC-MS determinations of ethanolic extracts (with the Soxhlet apparatus); Table S3: The comparison of the antioxidant activities of ethanolic extracts evaluated with the DPPH and ABTS methods; Table S4: The descriptive statistics of the antioxidant potentials of extracts measured with the DPPH method as TEAC values (mg/g); Table S5. The descriptive statistics of the antioxidant potentials of extracts measured with the ABTS method as TEAC values (mg/g); Table S6: The pH values of the obtained emulsions; Figure S1: Emulsions containing Olivem 1000 with different amounts of extracts: 1—base emulsion (without extract). With ethanolic extracts of the following plant materials: 2—dried *Rosmarinus officinalis* leaves, 3—dried *Salvia officinalis* L. leaves, 4—dried ground *Pinus sylvestris* L. cones; Figure S2: Emulsions containing glycerol monostearate as emulsifier: 1—base emulsion (without extract), 2—emulsion with extract of dried *Rosmarinus officinalis* leaves, 3—emulsion with extract of dried *Salvia officinalis* L. leaves, 4—emulsion with extract of dried ground *Pinus sylvestris* L. cones; Supplementary Equation (S1): %RSA versus Trolox concentration; Supplementary Equation (S2): Absorbance versus Trolox concentration.
