# Supplementary material for: Antioxidant Activities of Ethanolic Extracts Obtained from α-Pinene-Containing Plants and Their Use in Cosmetic Emulsions
Source: Antioxidants (Basel). 2024 Jul 4;13(7):811. doi: 10.3390/antiox13070811 (PMC11274233; doi:10.3390/antiox13070811)
Supplement: Supplementary file 1 [file antioxidants-13-00811-s001.zip › antioxidants-3061219-supplementary.pdf]

Supplementary Figures:

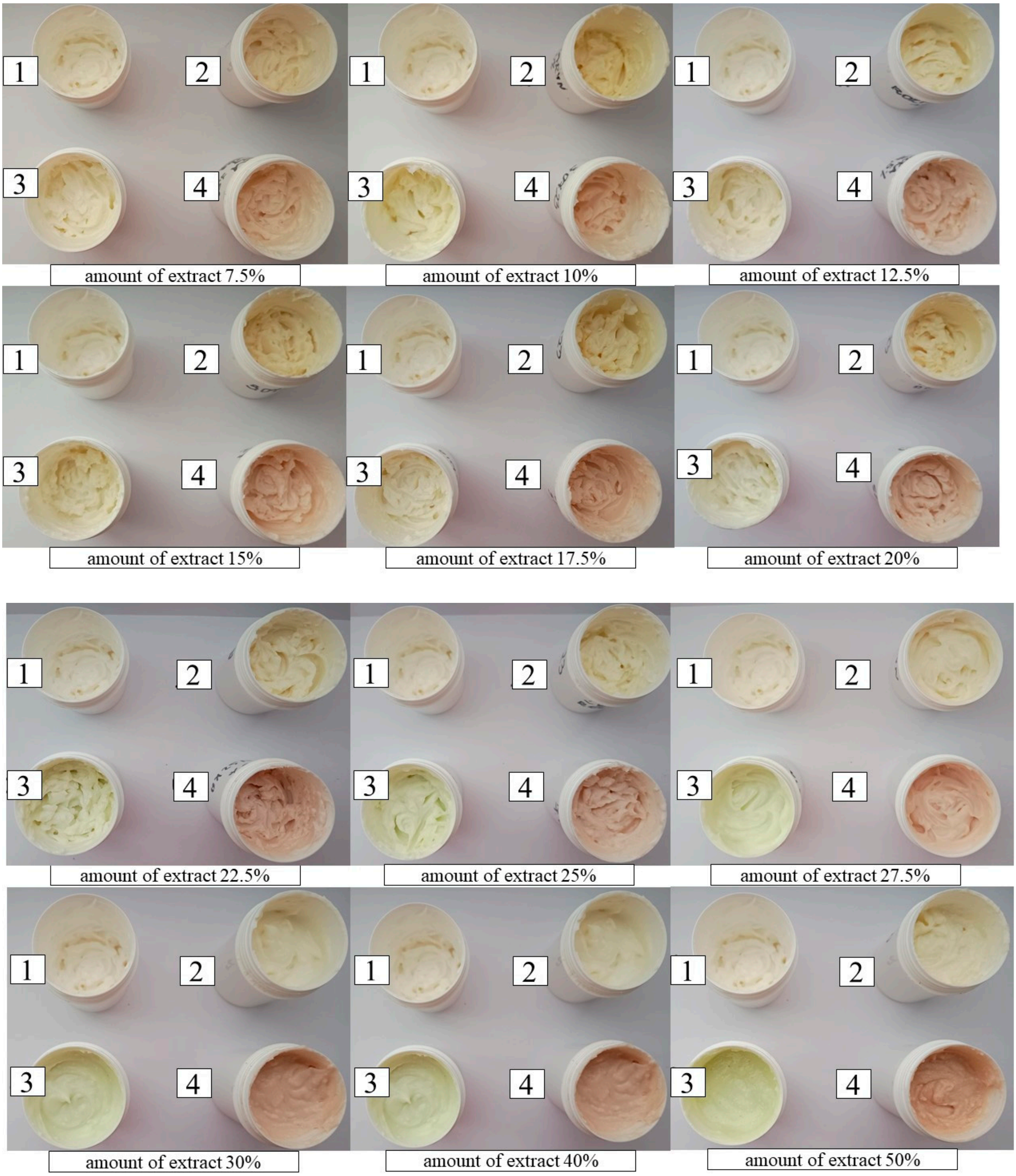

Figure S1. Emulsions containing Olivem 1000 with different amounts of extract: 1 – base emulsion (without extract). With ethanolic extracts of the following plant materials: 2 – dried *Rosmarinus officinalis* leaves, 3 – dried *Salvia officinalis* L. leaves, 4 – dried ground *Pinus sylvestris* L. cones

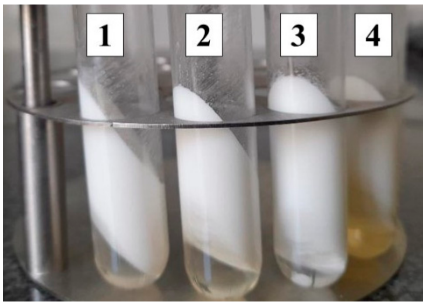

Figure S2. Emulsions containing glycerol monostearate as emulsifier: 1 - base emulsion (without extract), 2 - emulsion with extract of dried *Rosmarinus officinalis* leaves, 3 - emulsion with extract of dried *Salvia officinalis* L. leaves, 4 - emulsion with extract of dried ground *Pinus sylvestris* L. cones

Supplementary Tables:

Table S1. GC-MS determinations of ethanolic extracts (with ultrasound-assisted extraction)

compound name	fPin.c [1] [%]	fPin.n [2] [%]	fPin.b [3] [%]	uPin.c [4] [%]	gPin.c [5] [%]	dRos.l [6] [%]	fRos.l [7] [%]	dLev.l [8] [%]	dSal.l [9] [%]
α-pinene	2.21	1.81	3.06	2.85	4.70	14.54	3.14	9.58	4.83
acetamide	-	-	-	-	-	-	-	1.18	-
acetic acid	-	-	-	-	2.67	1.05	1.62	1.13	2.79
androstane-11	3.64	3.52	1.06	2.12	2.08	2.97	1.42	1.10	2.17
β-o-*cimene*	-	-	-	-	-	-	0.86	-	-
β-caryophyllene	-	-	-	-	-	1.53	-	-	-
bicyclo[3.1.1]heptane	-	-	-	1.66	-	0.99	2.79	-	-
bicyclo[2.2.1]hept-2-en-7-ol	-	-	-	-	-	-	-	-	2.00
borneol	-	-	-	-	-	11.44	-	-	-
camphor	1.72	1.58	1.48	2.24	1.39	9.23	2.25	3.39	3.93
carbon monoxide			2.28	3.11	1.00	0.72	3.01	1.00	5.17
cholestan-3-ol	-	-	-	-	-	-	4.25	2.88	4.63
2-cyclohexen-1-ol	-	-	-	-	-	-	-	0.83	-
2-cyclohexen-1-one	-	-	-	-	-	-	-	1.45	1.02
5,6-diamino-2,4-dihydroxypyrimidine	-	-	-	-	-	-	-	-	0.87
1,2-dihydro-2,3-diphenyl-quinazoline	-	-	-	-	-	-	-	2.59	-
1,4-dioxaspiro[4.5]decane	-	-	-	-	-	-	-	1.77	-
docosanoic acid	-	-	-	-	-	-	-	2.02	1.53
D-limonene	-	-	-	-	-	-	-	14.25	
epipowelline	-	-	-	-	-	-	2.24	2.15	1.76
eucalyptol	2.07	1.01	1.43	1.51	6.49	31.09	6.56	3.43	1.87
l-gala-l-ido-octose	-	-	-	-	-	-	-	1.79	1.46
glycolic acid	-	-	-	-	-	-	3.01	-	-
6H-benzofuro(3,2-c)(1)benzopyran	-	-	-	-	-	-	-	1.07	-
1H-cyclopenta[c]furan-3(3aH)-one	-	-	-	-	-	-	3.59	-	2.53
isobornyl thiocyanatoacetate	-	-	-	-	-	-	-	2.43	-
limonene oxide	-	-	-	-	-	-	-	0.63	-
7-(1-methylethylidene)bicyclo(4.1.0)heptane	-	-	-	-	-	2.55	-	-	-
nickel tetracarbonyl	-	-	-	-	2.74	1.05	-	3.26	1.09
nitrogen	-	-	-	-	-	-	2.30	2.51	5.37
10,13-octadecadienoic acid	-	-	-	-	-	-	1.42	-	-
pregnan-20-one	-	-	-	-	-	-	-	-	1.90
9,10-secocholesta-5,7,10 (19)-triene-3,24,25- triol	-	-	-	-	2.85	2.18	-	-	2.02
spiro[5-α-androstane-3,2'-thiazolidine]	-	-	-	-	-	1.27	-	-	-
tetradecane	-	-	-	-	-	-	-	1.06	-
3-thujen-2-ol	-	-	-	-	-	0.56	-	-	-
3-thujen-2-one	-	-	-	-	-	0.55	-	-	-
2-trifluoroacetoxydodecane	-	-	-	-	-	-	-	-	1.92
verbenone	-	-	-	-	1.53	0.55	3.46	2.61	1.61
viridiflorol	-	-	-	-	-	-	-	-	1.82
xanthatin	-	-	-	-	-	-	-	-	3.37

[1]fresh *Pinus sylvestris* L. cones, [2]fresh *Pinus sylvestris* L. needles, [3]fresh *Pinus sylvestris* L. branches, [4]dried unground *Pinus sylvestris* L. cones, [5]dried ground *Pinus sylvestris* L. cones, [6]dried *Rosmarinus*

officinalis leaves, [7]fresh *Rosmarinus officinalis* leaves, [8]dried *Levisticum officinale* leaves, [9]dried *Salvia officinalis* L. leaves

Table S2. GC-MS determinations of ethanolic extracts (with the Soxhlet apparatus)

compound name	dRos.l[1] [%]	dSal.l[2] [%]	gPin.c[3] [%]
α-pinene	2.21	2.85	1.01
acetamide	1.02	-	-
androstane-11	2.02	1.07	0.03
camphor	2.04	0.17	0.02
carbon monoxide	-	0.04	-
eucalyptol		0.06	0.11
isobornyl thiocyanatoacetate	1.02	-	-
nickel tetracarbonyl	-	0.03	0.03
3-thujanone	-	0.11	-
verbenol	1.06	-	-
verbenone	1.05	1.02	0.06

[1]dried *Rosmarinus officinalis* leaves, [2]dried *Salvia officinalis* L. leaves, [3]dried ground *Pinus sylvestris* L. cones

Table S3. Comparison of antioxidant activities of ethanolic extracts evaluated by the DPPH and ABTS methods

<table>
<tr><th>Raw material</th><th>Method</th><th>Ethanol concentration [%(v/v)]</th><th>Extraction time [min]</th><th>TEAC [mg/g]</th></tr>
<tr><td>dried Rosmarinus officinalis leaves</td><td rowspan="3">DPPH[a]</td><td>40</td><td>30</td><td>8.81</td></tr>
<tr><td>dried Salvia officinalis L. leaves</td><td>70</td><td>60</td><td>17.41</td></tr>
<tr><td>dried Rosmarinus officinalis leaves</td><td>96</td><td>30</td><td>9.69</td></tr>
<tr><td>dried Salvia officinalis L. leaves</td><td rowspan="3">ABTS[a]</td><td>40</td><td>60</td><td>50.60</td></tr>
<tr><td>dried Salvia officinalis L. leaves</td><td>70</td><td>60</td><td>36.37</td></tr>
<tr><td>dried ground Pinus sylvestris L. cones</td><td>96</td><td>60</td><td>15.04</td></tr>
<tr><td>dried Rosmarinus officinalis leaves</td><td>DPPH[b]</td><td>96</td><td>60</td><td>28.53</td></tr>
<tr><td>dried Rosmarinus officinalis leaves</td><td>ABTS[b]</td><td>96</td><td>60</td><td>8.63</td></tr>
</table>

[a]Ultrasound-assisted extraction, [b]Soxhlet apparatus extraction

Table S4. Descriptive statistics of antioxidant potentials of extracts measured by the DPPH method as TEAC values (mg/g)

Variables (n=27)	Min.	Max.	Mean	Standard deviation	Median	Lower quartile	Upper quartile
fresh *Pinus sylvestris* L. cones	0.987	3.707	2.219	0.787	2.260	1.558	2.595
fresh *Pinus sylvestris* needles	0.902	6.583	2.510	1.433	2.084	1.663	3.126
fresh *Pinus sylvestris* branches	1.738	11.052	5.015	2.961	3.793	2.179	6.919
dried unground *Pinus sylvestris* cones	2.610	3.602	3.236	0.245	3.312	3.121	3.392
dried ground *Pinus sylvestris* cones	3.061	3.667	3.367	0.145	3.372	3.287	3.447
dried *Rosmarinus officinalis leaves*	3.758	12.360	8.702	2.124	8.647	7.891	10.045
fresh *Rosmarinus officinalis* leaves	2.991	3.622	3.317	0.150	3.347	3.216	3.417
dried *Levisticum officinale* leaves	0.696	3.863	2.801	1.338	3.602	1.037	3.808
dried *Salvia officinalis* leaves	2.014	18.181	7.209	5.666	3.672	3.512	13.287

Table S5. Descriptive statistics of antioxidant potentials of extracts measured by the ABTS method as TEAC values (mg/g)

Variables (n=27)	Min.	Max.	Mean	Standard deviation	Median	Lower quartile	Upper quartile
fresh *Pinus sylvestris* L. cones	2.094	11.448	5.897	2.370	5.541	4.058	7.821
fresh *Pinus sylvestris* needles	2.715	10.265	5.681	2.149	5.421	3.798	7.520
fresh *Pinus sylvestris* branches	4.198	13.868	9.218	2.966	9.384	6.583	11.688
dried unground *Pinus sylvestris* cones	3.858	27.936	12.796	5.972	11.388	9.183	15.511
dried ground *Pinus sylvestris* cones	6.743	34.103	20.396	8.808	16.613	14.709	30.095
dried *Rosmarinus officinalis leaves*	6.603	15.691	10.766	2.545	10.406	8.562	13.312
fresh *Rosmarinus officinalis* leaves	4.659	24.329	12.164	5.210	13.788	6.343	15.812
dried *Levisticum officinale* leaves	1.693	33.221	14.800	11.162	12.550	2.936	25.611
dried *Salvia officinalis* leaves	4.780	53.086	27.294	17.816	25.932	6.222	42.991

Table S6. pH values of the obtained emulsions

Sample	amount of extract [%]	pH
base emulsion	0	4.63
emulsion with extract of dried *Rosmarinus officinalis* leaves	7.5	4.82
	10	4.88
	12.5	4.91
	15	4.97
	17.5	4.97
	20	5.01
	22.5	5.45
	25	5.51
emulsion with extract of dried *Salvia officinalis* L. leaves	7.5	4.90
	10	4.97
	12.5	4.99
	15	5.03
	17.5	5.04
	20	5.05
	22.5	5.44
emulsion with extract of dried ground *Pinus sylvestris* L. cones	7.5	4.76
	10	4.78
	12.5	4.82
	15	4.89
	17.5	4.92
	20	5.11
	22.5	5.20

Supplementary Equation (S1). %RSA versus Trolox concentration
- DPPH: $y = 95.23x + 5.2677$, $R^2 = 0.9961$, $R = 0.9980$
- ABTS: $y = 24.866x + 2.2999$, $R^2 = 0.9948$, $R = 0.9974$

Supplementary Equation (S2). Absorbance versus Trolox concentration
- DPPH: $y = -0.9502x + 0.9391$, $R^2 = 0.9978$; $R = 0.9989$
- ABTS: $y = -0.2502x + 0.9858$, $R^2 = 0.9954$; $R = 0.9977$
